# A Sensitive and Selective Label-Free Electrochemical DNA Biosensor for the Detection of Specific Dengue Virus Serotype 3 Sequences

**DOI:** 10.3390/s150715562

**Published:** 2015-07-01

**Authors:** Natália Oliveira, Elaine Souza, Danielly Ferreira, Deborah Zanforlin, Wessulla Bezerra, Maria Amélia Borba, Mariana Arruda, Kennya Lopes, Gustavo Nascimento, Danyelly Martins, Marli Cordeiro, José Lima-Filho

**Affiliations:** 1 Laboratório de Imunopatologia Keizo Asami (LIKA), Universidade Federal de Pernambuco-UFPE, Av. Prof. Moraes Rego, s/n, Campus da UFPE, 50670-901 Recife, PE, Brazil; E-Mails: daniellylsantos@hotmail.com (D.F.); deborahzanforin@gmail.com (D.Z.); wessullas@yahoo.com.br (W.B.); mariameliaborba@gmail.com (M.A.B.); mariana.s.arruda@gmail.com (M.A.); galvesn23@gmail.com (G.N.); bruneska@prospecmol.org (D.M.); joseluiz60@mac.com (J.L.-F.); 2 Universidade Federal de Alagoas (UFAL), Campus Arapiraca, Av. Manoel Severino Barbosa, s/n, Bom Sucesso, 57.309-005 Arapiraca, AL, Brazil; E-Mail: elainevms@yahoo.com.br; 3 Departamento de Virologia e Terapia Experimental (LAVITE), Centro de Pesquisas Aggeu Magalhães (CPqAM), Fundação Oswaldo Cruz (Fiocruz)—Pernambuco, Av. Professor Moraes Rego, s/n, Campus da UFPE, 50.670-420 Recife, PE, Brazil; E-Mail: kennya.genne@bol.com.br; 4 Departamento de Bioquímica, Universidade Federal de Pernambuco-UFPE, Av. Professor Moraes Rego, s/n, Campus da UFPE, CEP: 50670-901 Recife, PE, Brazil; E-Mail: Marli@cpqam.fiocruz.br

**Keywords:** dengue fever, DNA biosensors, differential pulse voltammetry, electrochemical detection, guanine oxidation

## Abstract

Dengue fever is the most prevalent vector-borne disease in the world, with nearly 100 million people infected every year. Early diagnosis and identification of the pathogen are crucial steps for the treatment and for prevention of the disease, mainly in areas where the co-circulation of different serotypes is common, increasing the outcome of dengue hemorrhagic fever (DHF) and dengue shock syndrome (DSS). Due to the lack of fast and inexpensive methods available for the identification of dengue serotypes, herein we report the development of an electrochemical DNA biosensor for the detection of sequences of dengue virus serotype 3 (DENV-3). DENV-3 probe was designed using bioinformatics software and differential pulse voltammetry (DPV) was used for electrochemical analysis. The results showed that a 22-m sequence was the best DNA probe for the identification of DENV-3. The optimum concentration of the DNA probe immobilized onto the electrode surface is 500 nM and a low detection limit of the system (3.09 nM). Moreover, this system allows selective detection of DENV-3 sequences in buffer and human serum solutions. Therefore, the application of DNA biosensors for diagnostics at the molecular level may contribute to future advances in the implementation of specific, effective and rapid detection methods for the diagnosis dengue viruses.

## 1. Introduction

Dengue fever is the most prevalent vector-borne disease in the world. The World Health Organization (WHO) estimates that some 100 million people are infected every year; however, some studies have predicted that this number could be greatly underestimated, and is actually closer to 390 million [1,2,3]. The distribution of the disease is mainly in tropical and subtropical regions and recently, it is has been increasingly seen in urban and semi-urban areas. All these factors have contributed to reveal dengue fever as a major international public health problem [1,2,4,5].

The infection is caused by a single stranded RNA-virus (DENV) of about 10.7 kb, which belongs to the *Flaviviridae* family, with approximately four antigenically distinct serotypes (DENV-1–DENV-4) [6,7]. The disease exhibits a wide range of symptoms, such as fever, headache and myalgia, which are the most common in classic dengue. Nevertheless, it can also shows more severe manifestations, like in dengue hemorrhagic fever (DHF) or dengue shock syndrome (DSS), which present life-threatening symptoms, such as bleeding, thrombocytopenia and vascular leakage [8,9,10].

Early diagnosis and identification of the pathogen are necessary for the prevention and treatment of patients, as well as for the avoidance of new outbreaks and emergence of severe cases of dengue, since it is known that the co-circulation of different serotypes in an area increases the possibility of DHF and DSS outcomes [11,12].

Methods to confirm dengue virus infection may involve detection of the virion, viral RNA, antigens or antibodies [13]. Virus detection by cell culture, viral nucleic acid or antigen detection (nonstructural protein 1 or NS1 antigen) can be used to confirm dengue infection in the acute phase of the illness (0–7 days following the onset of the symptoms) [14,15]. In the later phase of the disease, serologic tests are more applied and preferred for diagnosis, as the sensitivity of virus isolation and antigen reactivity decreases [16]. Viral antigen (NS1) detection assays are rapid, reliable and easy to perform, however, they cannot allow to distinguish between different viral serotypes [17,18].

Viral isolation, although considered the gold standard diagnostic method, is time-consuming and highly complex compared with other direct virus detection techniques [1,19]. On the other hand, the RT-PCR assay is widely used, it allows the detection of low copies of viral genes in less than 48 h [20]. However, both techniques are costly and labor-intensive, but they are more specific than serologic methods used for antibody detection and allow one to differentiate between the various dengue virus serotypes [21].

Application of DNA biosensors has emerged as an alternative method to the current molecular biology techniques [22,23]. These devices consist of a single-stranded DNA molecule (ssDNA) attached to a transducing surface that is able to detect a specific nucleic acid sequence, based on DNA hybridization events. Currently, there is a growing interest in developing label-free methods for DNA detection, considering their rapidness, easiness, low cost and minimal sample preparation requirements, compared to labeling methods, where the properties of the modified macromolecules often change, which may result in total loss of bioactivity or stability [24,25]. Label-free approaches rely on the direct detection of intrinsic electrochemical properties of DNA (e.g., oxidation of purine bases, particularly guanine) or on changes in some of the interfacial properties after hybridization. In addition, interference with the biological recognition between DNA molecules is minimized. Nevertheless, in labeling methods, these undesirable effects are more likely to occur due to steric hindrance and blocking of the binding sites [26,27,28].

Consequently, since biosensors allow to detect and identify DNA sequences in a fast and simple way, herein we report the first step to develop a cost-effective, sensitive and label-free electrochemical DNA biosensor for the detection of DENV-3 sequences in biological samples, as a part of an ongoing research previously published [29].

## 2. Experimental Section

### 2.1. Design of a Specific DENV-3 DNA Probe

The complete genomes of dengue virus serotype 3, corresponding to GenBank accession numbers AY099336, AY099337, AY099338S1,AY099338S2, AY099339S1, AY099339S2, AY099340S1, AY099340S2, AY09934S1, AY099342S1 were obtained from the National Center for Biotechnology Information (NCBI) database. These sequences correspond to strains that were introduced in the American continent, and caused the disease outbreaks in 2002 [30,31]. CLC Main Workbench v.6.0 software was used to analyze common sequences among those dengue genomes, by using an alignment tool. Then, a specific DNA probe for DENV-3 was selected by comparison of the homologous sequences with other organisms, using Basic Local Alignment Search Tool (BLAST). DENV-3 complementary (target) and non-complementary sequences were also designed using the same method.

### 2.2. Reagents and Materials

All chemicals were of reagent grade quality and were used directly as received without further purification. Tris base was obtained from Promega (Fitchburg, WI, USA) and sodium acetate was obtained from Sigma-Aldrich (St. Louis, MO, USA) DENV-3 probes were purchased as lyophilized powder from IDT Technologies (Coraville, IA, USA). The stock and diluted solutions (25 nM) were prepared in 0.5 M acetate buffer (pH 5.0) and kept frozen. Ultrapure RNAse/DNAse-free water was used in all buffer solutions. After bioinformatics analysis, the following DNA sequences were used in this study:

DENV-3 probe: 5′-TAA CAT CAT CAT GAG ACA GAG C-3′
DENV-3 target: 5′-GCT CTG TCT CAT GAT GAT GTT A-3′
Non-complementary sequence: 5′-TCT CTT GTT TAA GAC AAC AGA G-3′

Human serum used in this study was obtained from blood samples provided by the pathogenic virus collection of Centro de Pesquisas Aggeu Magalhães (CPqAM). Serum solutions were prepared by centrifugation at 3500 rpm for 5 min at 20 °C (3500 rpm for 5 min), in which the obtained supernatant was collected from each sample, and stored at 23 °C until further used for experiments testing.

### 2.3. Apparatus

Experiments were carried out using a PGSTAT302 potentiostat (METROHM Autolab, Utrecht, The Netherlands) with the GPES 4.9.007 software as a graphic interface. The electrochemical device was composed by a two-electrode system: A pencil graphite electrode (PGE) as a working electrode and silver/chloride silver electrode as a reference electrode. Each measurement consisted of a cycle of activation/immobilization/hybridization/detection by using a fresh PGE surface. All the experiments were performed in triplicate, at room temperature (23 °C).

### 2.4. Procedure

#### 2.4.1. Preparation of Electrodes and Pre-Treatment of PGE

PGEs were obtained from Mercur (Santa Cruz do Sul, Brazil), as a pencil graphite lead type 4 B. Briefly, PGEs were produced by cutting graphite lead in pieces of 3 cm and polishing them with an emery polishing disc (Dremel, Mount Prospect, IL,USA). The PGEs were then washed with ultrapure water to remove any contaminant present on the surface of the working electrode. The reference electrode was made by immersing a golden pin into an Ag/AgCl ink (Henkel Acheson, Hemel Hempstead, UK) and dried at 40 °C overnight. The polished surface of PGEs was pre-treated by applying a potential of +1.80 V for 5 min in 0.5 M acetate buffer solution (pH 5.0) [32,33,34].

#### 2.4.2. DNA Probe Immobilization onto PGE Surface

Immobilization of DENV-3 probe onto the PGE surface was performed by immersing the activated PGE in acetate buffer solution, with different concentrations of DENV-3 probes (250–1000 nM), by applying a fixed potential of +0.5 V for 300 s onto the electrode surface.

#### 2.4.3. DNA Hybridization with Complementary and Non-Complementary Sequences

The hybridization of the immobilized DNA probe on the electrode (PGE/DENV-3 probe) was performed by immersing the electrode in an Eppendorf tube containing 70 μL of DENV-3 target sequences diluted in acetate buffer. The hybridization reaction was then carried out in a thermomixer, stirring at 300 rpm, under a specific annealing temperature of 52 °C, for 10 min. This same procedure was adopted to evaluate the hybridization of the PGE/DENV-3 probe with non-complementary sequences, as well as buffer solutions containing a mix of both 75 nM of DENV-3 complementary and non-complementary sequences (mixed DNA solution).

#### 2.4.4. Detection of Complementary and Non-Complementary Sequences in Human Serum

As a way to evaluate the efficiency of the system to detect DENV-3 sequences in biological samples on the electrode surface, the complementary and non-complementary DNA sequences were diluted in human serum (75 nM concentration) and the hybridization assay was conducted using the same conditions described previously. This procedure was also adopted for tests human serum solutions mixed with both target and non-complementary sequences.

### 2.5. Electrochemical Analysis

Differential pulse voltammetry (DPV) was used for electrochemical analysis in this study. Current peaks were recorded after applying a potential range of +0.5 up to +1.2 V at a scan rate of 0.05 V/s onto the electrode surface, which was immersed in 20 mM Tris-HCl buffer (pH 7.0). The raw data obtained with DPV technique was treated using the Savitzky and Golay filter (level 2) of the GPES software, followed by moving the average baseline correction using a peak width of 0.01 V [35].

### 2.6. Statistical Data Analysis

Experimental data were analyzed with Statistica 8.0 software (StatSoft, Tulsa, OK, USA) using parametric tests; Tukey’s test was used to compare multi-independent group data, and a level of *p* < 0.05 was considered significant. The reproducibility of the system was expressed as the coefficient of variation inter-assay (CV), which was calculated over three independent assays on the probe-modified PGEs.

## 3. Results and Discussion

### 3.1. Bioinformatics Analysis of DENV-3 DNA Probes

**Figure 1:**
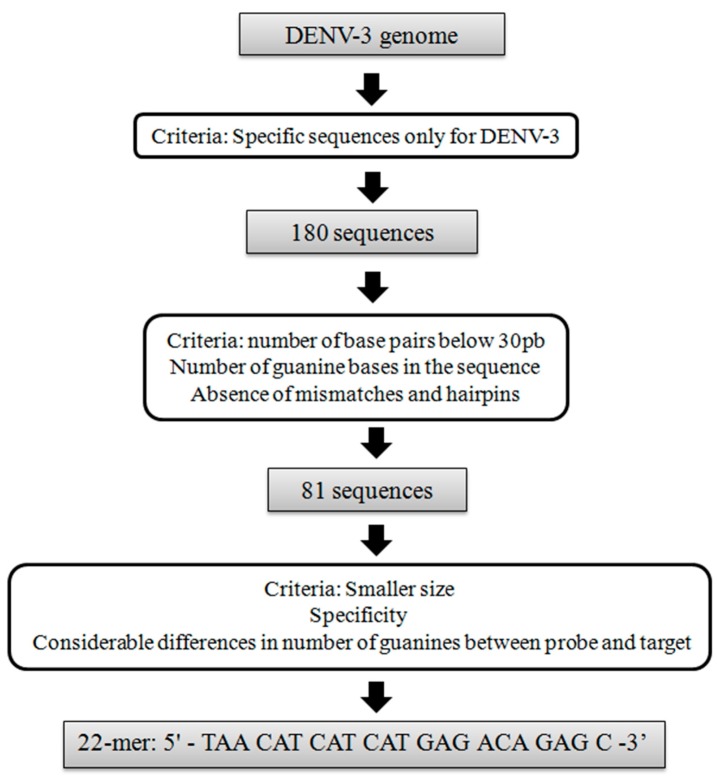
Flowchart of the selection criteria used to design the DENV-3 probe.

The design of DNA probes is one of the crucial steps in the development of a biosensor, because it determines the specificity of the device [36]. For genosensors, this can be achieved using bioinformatics analysis based on whole genome sequencing, in a way to predict the most specific region that is able to produce a steady double-strand DNA with the pathogen [37,38]. In this work, DNA probes specific for DENV-3 were designed mainly by using CLC Main Workbench software, based on a sequence alignment tool to identify regions of similarity between the dengue strains. After that, DNA sequences from the strains that showed specificity only for DENV-3 were compared with other organism genomes using BLAST tool, in order to exclude any correlations. Finally, the Oligonucleotide Properties Calculator (Oligo Calc) software (Northwestern University, IL, USA) was used to provide physical properties information of the selected DENV-3 sequences, in a way to establish the best match of DNA probe for biosensors. Figure 1 shows a flowchart containing the criteria of selection of DENV-3 probes used in this study. 

First, it was shown that 180 DNA sequences were specific only for dengue virus serotype 3. Among these, 81 were selected as DNA probes, based on the number of base pairs of the sequence, number of guanine bases and the absence of mismatches and hairpins. Finally, a 22-m oligonucleotide was selected to detect DENV-3, with the following sequence: 5′-TAA CAT CAT CAT GAG ACA GAG C-3′. This sequence was selected due to its suitable features that are desirable for electrochemical biosensors, such as shorter base pair length, high specificity and a considerable difference in the number of guanine base between the probe and target sequences, which is important to discriminate between ssDNA and dsDNA onto the PGE surface [33,39,40].

In addition, this probe was targeted to detect sequences from the envelope (E) gene, which is responsible for binding and fusion to host cell membranes [4,41]. This particular gene was chosen because of it is highly conserved sequence, which suffers less mutation process rather than other parts of dengue genome. Viral gene regions that interact with specific host cells are evolutionarily constrained, mainly in viruses that infect multiple organisms, like dengue virus. This is important to be considered in the development of DNA biosensors to detect dengue virus, once that it determines the selectivity and specificity of the method, avoiding cross-reactivity with non-related organisms [42,43,44].

### 3.2. Effect of DENV-3 Probes Concentration on Immobilization on the PGE

The immobilization of a biological element on the electrode is the first step to be considered in the development of a biosensor [45]. Determination of the optimal probe concentration is crucial to ensure a high performance of DNA biosensors, and reduce any interference in the electrochemical response of the system [46,47]. Thus, the effect of DENV-3 probes concentration was also investigated in this study.

**Figure 2:**
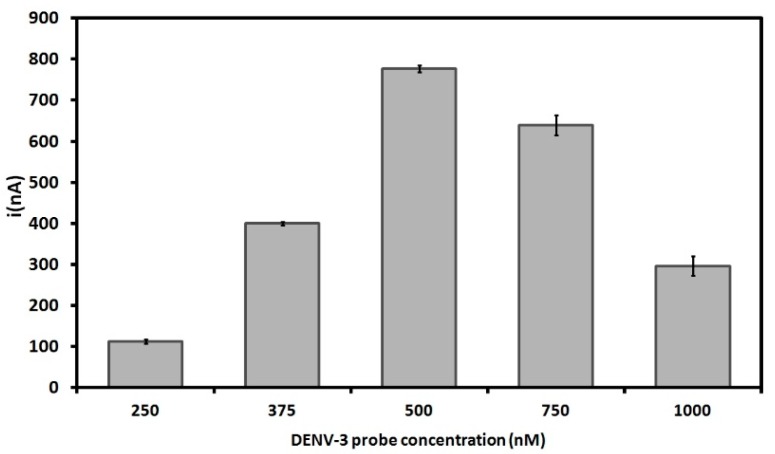
Electrochemical signals of different concentrations of DENV-3 probe onto pencil graphite electrodes (PGE). Differential pulse voltammetry (DPV) was used for electrochemical analysis based on guanine oxidation. Experimental conditions: Scanning potential range between +0.5 V and +1.2 V and scan rate of 0.05 V/s. The results represent the average of triplicates carried out at each DENV-3 probe concentration.

Figure 2 shows current peaks of different DENV-3 probe concentrations on the PGE surface. As the electrochemical analysis in this study relies on label-free oxidation of guanine bases, the acquisition of higher current signals for DNA probes is well-suited for this system [29,48,49,50]. The results show that the current gradually rises with the increase of the probe concentration from 250 nM up to 500 nM, reaching the highest electrochemical signal of 777 ± 8.6 nA at 500 nM. The result obtained at 500 nM was also statistically different from that obtained at 750 nM (*p* = 0.000178). However, the decrease in the current peaks at higher concentrations of DNA probes after 500 nM could be due to the steric hindrance between the nitrogenous bases and the transducer. This prevents the electrons produced by the oxidation process to access the electrode surface [51,52,53,54,55]. Therefore, a concentration of 500 nM was selected as the optimal probe concentration for DNA immobilization on the PGE.

### 3.3. Electrochemical Analysis of Hybridization Assays

**Figure 3:**
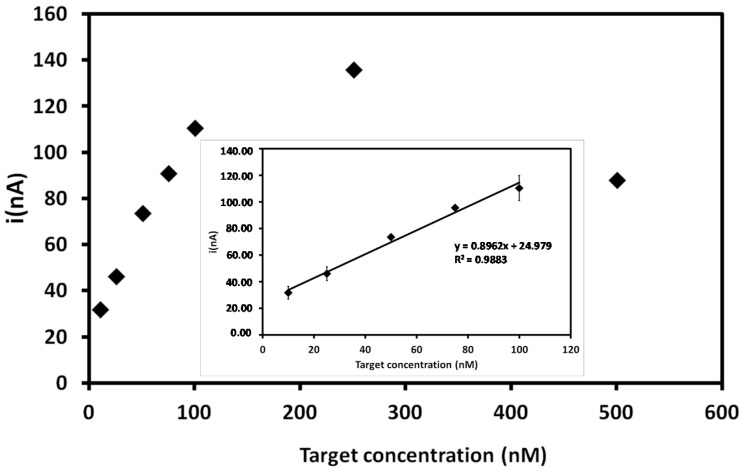
Current signals obtained for different DENV-3 target sequence concentrations after hybridization with probe-modified PGEs. Inset: Related calibration graph at a concentration range of 10–100 nM for the target sequence. Experimental conditions: Scanning potential range between +0.5 V and +1.2 V and scan rate of 0.05 V/s.

In this study, the biosensor performance was analyzed through the hybridization reaction between the DENV-3 probe and the complementary DENV-3 oligonucleotide. Hybridization was carried out with different amounts of the target sequence and this reaction was performed in an electrochemical cell containing 20 mM Tris-HCl buffer (pH 7.0). The electrochemical signals based on guanine oxidation are displayed in Figure 3. The results showed that the current peaks increase with the increasing concentration of the target sequence (10 nM to 500 nM); the highest concentration exhibited the highest current peak of the system (135 ± 2.15 nA). However, at concentrations higher than 500 nM, there is a decrease in the electrochemical signal that could be due to electrostatic hindrance of DNA molecules on the PGE surface [39,53,56].

The linear regression of the current peaks obtained from different concentrations of DENV-3 target is shown in the inset of Figure 3. The calibration curve (described by the equation *y* = 0.8962*x* + 24.979) is linear between 10 nM and 100 nM, with a correlation coefficient of 0.9883 (*p* < 0.00536, *n* = 5). A detection limit of 3.09 nM could be estimated with the following equation: 3 *s*/*m*, where *s* is the standard deviation of most reproducible current peak result (corresponding to 75 nM concentration) and *m* is the slope of the linear regression [57]. The same experimental conditions were used to estimate the reproducibility of the method, which was 1.01%, indicating, thus, the significant reproducibility of the method.

**Figure 4:**
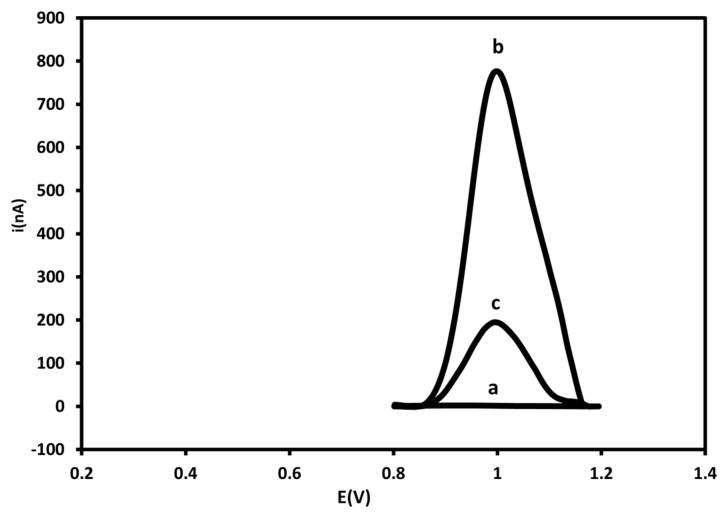
Differential pulse voltammograms corresponding to bare PGE (**a**), probe-modified PGE before (**b**) and after hybridization with 250 nM of target sequence (**c**) in 20mM Tris-HCl buffer solution (pH 7.0). Experimental conditions: Scanning potential range between +0.5 V and +1.2 V and scan rate of 0.05 V/s.

Figure 4 displays electrochemical signals of probe-modified PGE before and after hybridization with 250 nM of the target. A decrease of 83% in the current signal was observed after the reaction with the DENV-3 target; this is due to the fact that oxidizable regions of guanine bases in the ssDNA (777 nA) are bound through hydrogen bonds that held the double chain together, thus decreasing the electrochemical signal of the dsDNA (135 nA) on the electrode surface [24,58,59,60].

Furthermore, as is seen in Table 1, the present sensor has a lower detection limit (3.09nM) compared to other electrochemical DNA biosensors.

**Table 1:** Comparison of the analytical performance of different electrochemical DNA biosensors.

Nucleic Acid Biosensor	Electrode	Electrochemical Method	Linear Range of Hybridization	Detection Limit	Reference
Single-walled carbon nanotubes-polymer modified graphite electrodes for DNA hybridization	PGE ^a^	DPV ^d^	50–200μg/mL	5.14 μM	[61]
Hybridization biosensor for detection of hepatitis B virus	GCE ^b^	DPV	0.36–1.32 μM	19.4 nM	[62]
Brilliant cresyl blue as electroactive indicator in electrochemical DNA oligonucleotide sensors	CPE ^c^	DPV	10 nM–5μM	9 nM	[63]
Label-free DNA detection based on zero current potentiometry	PGE	LSV ^e^	10 nM–1μM	6.9 nM	[64]
DNA biosensor detection of DENV-3 sequences onto PGE surfaces	PGE	DPV	10–100 nM	3.09 nM	This work

^a^ Pencil graphite electrode; ^b^ Glassy carbon electrode; ^c^ Carbon paste electrode; ^d^ Differential pulse voltammetry; ^e^ Linear sweep voltammetry.

### 3.4. Selectivity Study

**Figure 5:**
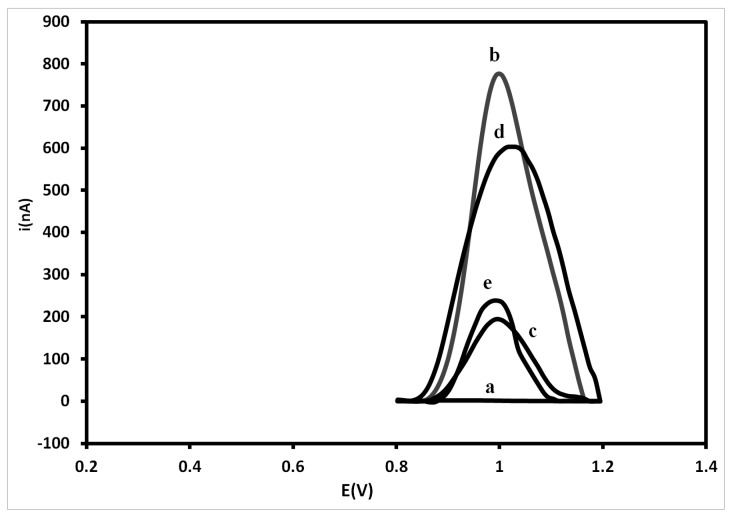
Differential pulse voltammograms for guanine oxidation of (**a**) bare PGE; (**b**) probe-modified PGE; (**c**) probe-modified PGE after hybridization with DENV-3 sequence; (**d**) non-complementary sequence and (**e**) a mixed solution of DENV-3 sequence and non-complementary sequence. Experimental conditions: Scanning potential range between +0.5 V and +1.2 V and scan rate of 0.05 V/s.

In a way to evaluate the selectivity of the DENV-3 biosensor, hybridization tests were performed with a non-complementary sequence. DPV voltammograms for bare PGE, probe-modified PGE before and after hybridization with DENV-3 target and non-complementary sequence are displayed in Figure 5. It was verified that no electrochemical signal was recorded with bare PGE, which is in agreement with the absence of DNA on the electrode surface. Probe-modified PGE presented the highest current peak of the system, whereas the probe-modified PGE after hybridization with target sequence showed a decrease in the current signal, as discussed previously.

As shown in Figure 5, a significant difference in the voltammetric signal was observed after hybridization of DENV-3 probe with the non-complementary sequence (600 nA) when compared with the complementary DNA (135 nA); however, the signal was slightly lower compared to the probe-modified electrode (777 nA). This may be attributed to some unspecific hybridization of non-complementary sequences with the probe. Nevertheless, the target sequence is clearly able to form a steady dsDNA on the electrode surface. Moreover, a decrease in the current peak was also noticed when the probe-modified PGE was added to the mixed DNA solution (~230 nA) when compared with the probe-modified electrode. Therefore, these results can confirm the ability of the PGE-modified biosensor to detect selectively dengue virus serotype 3 [34,61,62].

### 3.5. Electrochemical Measurement of Target Hybridization in Human Serum Solutions

In order to evaluate the efficiency of the probe surface for biosensing applications and in an attempt to test the performance if the biosensor for the detection DENV-3 in real samples, DPV was used to investigate DNA hybridization on PGE surface using human serum. This assay was tested with 75 nM of target sequence, non-complementary sequences and a solution mixed of both target and non-complementary sequences.

**Figure 6:**
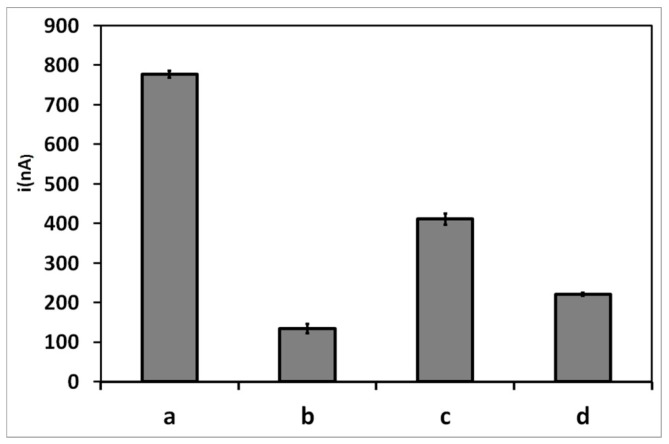
Current peaks related to guanine oxidation of the probe-modified-PGE after (**a**) and before hybridization with DENV-3 (**b**); in the presence of non-complementary sequences (**c**) and in a solution mixed with DENV-3 and non-complementary sequences (**d**), all diluted in human serum. Experimental conditions: Scanning potential range between +0.5 V and +1.2 V and scan rate of 0.05 V/s.

As shown in Figure 6, the biosensor displays the same electrochemical behavior observed previously in Tris-HCl buffer solutions. However, all the current signals of the probe-modified PGE after hybridization with the complementary, non-complementary sequences and mixed DNA solution diluted in human serum presented a slight decrease (134, 410.8 and 221 nA, respectively) when compared with those diluted in acetate buffer (135, 600 and 230 nA, respectively). This could be due to the hybridization kinetics and the efficiency of the PGE surface, which could be affected by non-specific adsorption of plasma proteins, and this may interfere with the detection of the electrochemical signal [63]. However, such interference with the detection of DNA molecules was observed previously with the optical DNA biosensor developed by Gong *et al.* [64,65]*.* Thus, these results confirm the high selectivity and sensitivity of the electrochemical DNA biosensor developed herein.

## 4. Conclusions

A sensitive DNA biosensor based on electrochemistry for the detection of dengue virus serotype 3 was proposed in the present study. A pencil graphite electrode, modified with a probe designed specifically for DENV-3, was able to identify selectively target sequences of the virus, with a low detection limit of 3.09 nM. Moreover, the probe-modified PGE allowed to detect specifically complementary sequences of the target DNA spiked with human serum. 

The sensitivity of this assay can be further improved by testing other electrode materials, such as gold, platinum and grapheme electrodes. In addition, screen-printed electrodes could be also used for the implementation of a portable system. Therefore, the application of biosensors for the diagnosis of dengue virus at the molecular level may contribute to the future development and advancement of effective and rapid detection methods.
